# Stomach Chitinase from Japanese Sardine *Sardinops melanostictus*: Purification, Characterization, and Molecular Cloning of Chitinase Isozymes with a Long Linker

**DOI:** 10.3390/md14010022

**Published:** 2016-01-20

**Authors:** Satoshi Kawashima, Hiroki Ikehata, Chihiro Tada, Tomohiro Ogino, Hiromi Kakizaki, Mana Ikeda, Hideto Fukushima, Masahiro Matsumiya

**Affiliations:** Department of Marine Science and Resources, College of Bioresource Sciences, Nihon University, Fujisawa, Kanagawa 252-0880, Japan; kawashima.tp@gmail.com (S.K.); mg7759@gmail.com (H.I.); mackerel@mrj.biglobe.ne.jp (C.T.); ogino19900829@yahoo.co.jp (T.O.); himibambi@hotmail.co.jp (H.K.); perfect-love@road.ocn.ne.jp (M.I.); fukushima.hideto@nihon-u.ac.jp (H.F.)

**Keywords:** fish stomach chitinase isozymes, cDNA cloning, Japanese sardine *Sardinops melanostictus*, fish-specific acidic fish chitinase, long linker

## Abstract

Fish express two different chitinases, acidic fish chitinase-1 (AFCase-1) and acidic fish chitinase-2 (AFCase-2), in the stomach. AFCase-1 and AFCase-2 have different degradation patterns, as fish efficiently degrade chitin ingested as food. For a comparison with the enzymatic properties and the primary structures of chitinase isozymes obtained previously from the stomach of demersal fish, in this study, we purified chitinase isozymes from the stomach of Japanese sardine *Sardinops melanostictus*, a surface fish that feeds on plankton, characterized the properties of these isozymes, and cloned the cDNAs encoding chitinases. We also predicted 3D structure models using the primary structures of *S. melanostictus* stomach chitinases. Two chitinase isozymes, SmeChiA (45 kDa) and SmeChiB (56 kDa), were purified from the stomach of *S. melanostictus*. Moreover, two cDNAs, *SmeChi-1* encoding SmeChiA, and *SmeChi-2* encoding SmeChiB were cloned. The linker regions of the deduced amino acid sequences of *SmeChi-1* and *SmeChi-2* (SmeChi-1 and SmeChi-2) are the longest among the fish stomach chitinases. In the cleavage pattern groups toward short substrates and the phylogenetic tree analysis, SmeChi-1 and SmeChi-2 were classified into AFCase-1 and AFCase-2, respectively. SmeChi-1 and SmeChi-2 had catalytic domains that consisted of a TIM-barrel (β/α)_8_–fold structure and a deep substrate-binding cleft. This is the first study showing the 3D structure models of fish stomach chitinases.

## 1. Introduction

Chitinases (EC 3.2.1.14)—enzymes that randomly hydrolyze the β-1,4 glycosidic bonds of chitin, a water-insoluble homopolymer composed of β-1,4-linked *N*-acetyl-d-glucosamine (GlcNAc)—are widely distributed in a variety of living organisms, and they have roles in various biological processes [1,2]. *N-*acetylchitooligosaccharides ((GlcNAc)_n_) and GlcNAc are hydrolysis products of chitin with a variety of physiological functions, such as immunostimulatory activity [3,4], improvement of skin quality and alleviate osteoarthritis [5,6], respectively. Therefore, chitinases are useful enzymes for enzymatic production of (GlcNAc)_n_ and GlcNAc. Chitinases are classified into glycosyl hydrolases (GH) families 18 and 19 based on the amino acid sequence similarity of their catalytic domains [7]. The catalytic domains of family 18 chitinases have a TIM-barrel (β/α)_8_-fold [8,9], whereas the catalytic domains of family 19 chitinases have a high α-helical content [10,11].

Regarding marine organisms, chitinases have been purified from the stomach of several Osteichthyes [12,13,14,15,16,17], the livers of Japanese common squid *Todarodes pacificus* [18] and golden cuttlefish *Sepia esculenta* [19], and the posterior salivary gland of common octopus *Octopus vulgaris* [20], and these chitinases have been characterized. Chitinases genes were cloned from the stomach of several Osteichthyes [16,17,21,22], the stomach of the Chondrichthyes blue shark *Prionace glauca* [23], the hepatopancreas of swimming crab *Portunus trituberculatus* [24], and the tissues of several shrimps [25,26]. Chitinase cDNA from the stomach of Coelacanths *Latimeria menadoensis* considered ancestors of the superclass tetrapoda, was also cloned and its expression profile and the results of a phylogenetic analysis were reported [27].

Fish chitinases are reported to have high activity in the stomach [12,13,14,15,16,17]. Fish stomach chitinases degraded crystalline α- or β-chitin and crystalline chitin nanofibers [13,14,15,16,17] with uniform widths of approximately 10–20 nm that were prepared from crab chitin flakes as gels and dissolved in water [28]. In particular, two chitinase isozymes (PtChiA and PtChiB) purified from the stomach of threeline grunt *Parapristipoma trilineatum* [16] and three chitinase isozymes (SmChiA, SmChiB, and SmChiC) purified from the stomach of the marbled rockfish *Sebastiscus marmoratus* [17] exhibited markedly high activity toward chitin nanofibers.

We reported that fish express two different chitinases, acidic fish chitinase-1 (AFCase-1) and acidic fish chitinase-2 (AFCase-2), in the stomach that have different degradation patterns, as fish efficiently degrade chitin ingested as food [16,17,22]. In particular, AFCase-1 preferentially hydrolyzes the second glycosidic bond from the non-reducing end of (GlcNAc)_n_, whereas AFCase-2 preferentially hydrolyzes the third glycosidic bond [13,14,15,16,17,22]. In addition, a phylogenetic tree analysis of vertebrate chitinases revealed that AFCase-1 and AFCase-2 form unique chitinase groups which differ from the chitotriosidases and acidic mammalian chitinase (AMCases) that are found in mammalian macrophages [29] and mammalian stomachs [30], respectively.

Regarding fish stomach chitinase, although the primary structure and the enzymatic properties of mainly fish stomach chitinases of demersal fish that feed on shrimp, crab, and squid have been elucidated, there is little information concerning the primary structure and the enzymatic properties of surface fish chitinases. There is also no information concerning 3D structure models of fish stomach chitinases.

In the present study, we aimed to purify chitinase isozymes from the stomach of Japanese sardine *Sardinops melanostictus*, a surface fish that feeds on plankton, and we characterized the properties of these isozymes. For a comparison with the primary structures of chitinase cDNAs obtained previously from the stomach of demersal fish [16,17,21,22], we cloned the cDNAs encoding chitinases from the stomach of *S. melanostictus* and determined their primary structures. We also predicted the 3D structure models using the primary structures of *S. melanostictus* stomach chitinases, and we compared them with those of other previously reported chitinases. To our knowledge, this is the first study showing the 3D structure models of fish stomach chitinases.

## 2. Results and Discussion

### 2.1. Purification of SmeChiA and SmeChiB

After ammonium sulfate fractionation, the enzyme solution was applied to a Chitin EX column, and chitinase activity was detected in both non-adsorbed and adsorbed fractions. The adsorbed fraction was further purified by cation exchange chromatography using a TOYOPEARL CM-650S column and obtained chitinase active fraction (SmeChiA). The non-adsorbed fraction was fractionated by anion exchange chromatography using a TOYOPEARL DEAE-650S column and further purified a TOYOPEARL CM-650S column and obtained chitinase active fraction (SmeChiB).

**Figure 1:**
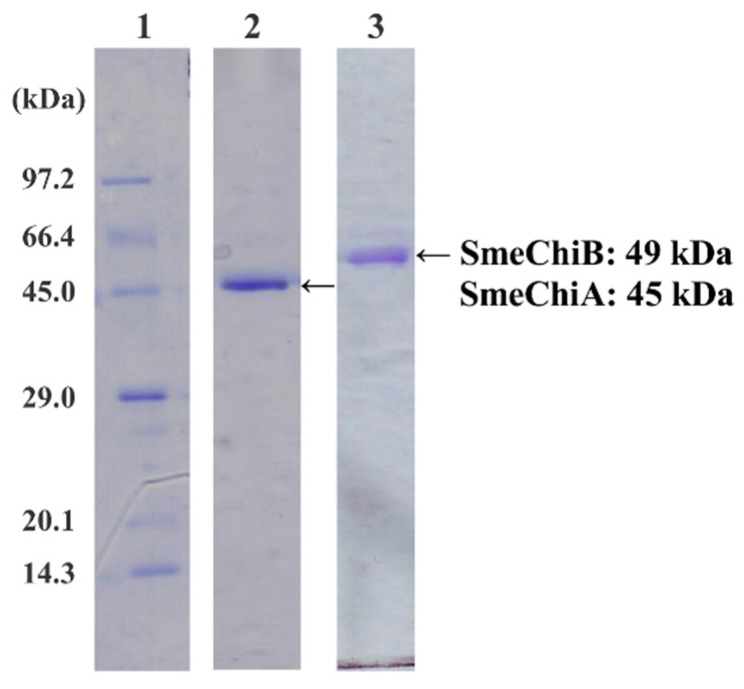
SDS-PAGE of SmeChiA and SmeChiB. Lane 1: marker proteins. The marker proteins used were phosphorylase b (97.2 kDa), serum albumin (66.4 kDa), ovalbumin (45.0 kDa), carbonic anhydrase (29.0 kDa), trypsin inhibitor (20.1 kDa), and lysozyme (14.3 kDa). Lane 2: SmeChiA. The amount of protein applied was 0.21 μg. Lane 3: SmeChiB. The amount of protein applied was 0.23 μg.

In sodium dodecyl sulfate-polyacrylamide gel electrophoresis (SDS-PAGE), purified chitinase isozymes, SmeChiA and SmeChiB, showed a single band; the apparent molecular masses were estimated to be 45 kDa and 49 kDa, respectively (Figure 1). When *p*-nitrophenyl (pNp)-(GlcNAc)_2_ was used as the substrate, the specific activities of SmeChiA and SmeChiB were 0.660 and 0.219 U/mg, and their purifications were 47.1 and 20.8-fold, respectively.

The N-terminal amino acid sequences of chitinase isozymes were analyzed up to the 25th residue. The N-terminal amino acid sequence of SmeChiA and SmeChiB showed Y L L S N Y F T N W G Q Y R P G A G K Y F P K D V and H I L S N Y F T N W A Q Y R P P P T I Y M P K D I, respectively, and thus SmeChiA and SmeChiB have different N-terminal amino acid sequences. These results demonstrated that *S. melanostictus* as well as silver croaker *Pennahia argentata* (PaChiA and PaChiB) [14,15] and *P. trilineatum* (PtChiA and PtChiB) [16] have two chitinase isozymes in stomach.

### 2.2. Effect of pH on SmeChiA and SmeChiB Activities

**Figure 2:**
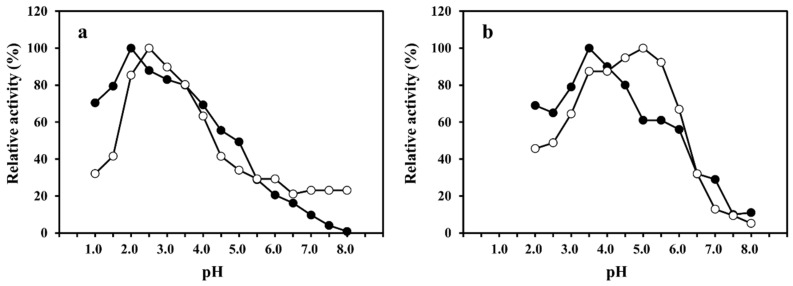
Effect of pH on the activities of SmeChiA and SmeChiB. The optimum pH of SmeChiA (**a**) and SmeChiB (**b**) using pNp-(GlcNAc)_2_ (●) and pNp-(GlcNAc)_3_ (○) as a substrate. Maximum activity was 100%.

The optimum pH values of SmeChiA toward pNp-(GlcNAc)_2_ and pNp-(GlcNAc)_3_ were observed to be pH 2.0 and pH 2.5 (Figure 2a), and in comparisons of the optimum pH values of PaChiA [14], SmChiA, SmChiB [17], and PtChiA [16], the maximum activity of SmeChiA toward pNp-(GlcNAc)_2_ and pNp-(GlcNAc)_3_ showed a stronger acid region. The optimum pH values of SmeChiB toward pNp-(GlcNAc)_2_ and pNp-(GlcNAc)_3_ were pH 3.5 and pH 5.0, respectively (Figure 2b). The optimum pH values of PaChiB [15] and PtChiB [16] for pNp-(GlcNAc)_3_ were reported to be 5.0, which is concordant with the value for SmeChiB.

When pNp-(GlcNAc)_3_ was used as a substrate, SmeChiB showed maximum activity at pH 5.0, whereas SmeChiA showed 34% or less of the maximum activity. As shown in Figure 2, SmeChiA worked well in the acidic pH range compared to SmeChiB. The relative activity toward pNp-(GlcNAc)_2_ and pNp-(GlcNAc)_3_ of both chitinases exhibited 30% or less of the maximum activity at pH 7.0.

These results demonstrated that the *S. melanostictus* stomach chitinases showed the optimum pH in the acidic region (Figure 2). It was reported that fish stomach chitinases exhibited activity in the acidic pH range [12,13,14,15,16,17]. This characteristic of SmeChiA and SmeChiB, as well as other fish stomach chitinases, is thought to be appropriate for a physiological role in the digestion of chitinous substances ingested as food in the presence of gastric acid [31].

### 2.3. Substrate Specificities of SmeChiA and SmeChiB toward pNp-(GlcNAc)_n_ (n = 1–4) and (GlcNAc)_5_

Although SmeChiA and SmeChiB released pNp from pNp-(GlcNAc)_n_ (*n* = 2–4), they did not degrade pNp-(GlcNAc) (Table 1). Therefore, SmeChiA and SmeChiB as well as other fish stomach chitinase [16,17,18,19,20,21] do not have β-*N*-acetylhexosaminidase activity. SmeChiA showed high activity (0.660 U/mg) toward pNp-(GlcNAc)_2_. The second highest activity of SmeChiA was toward pNp-(GlcNAc)_3_ (0.081 U/mg), but its activity toward pNp-(GlcNAc)_4_ was markedly lower. SmeChiB showed high activity (0.877 U/mg) toward pNp-(GlcNAc)_3_, approximately 11-fold higher than that shown by SmeChiA (Table 2).

**Table 1:** Substrate specificities of SmeChiA and SmeChiB toward pNp-(GlcNAc)_n_ (*n* = 1–4).

Substrate	Specific Activity (U/mg)	
	SmeChiA	SmeChiB
*p*-nitrophenyl-(GlcNAc) (G-P)	ND	ND
*p*-nitrophenyl-(GlcNAc)_2_ (G-G-P)	0.660	0.219
*p*-nitrophenyl-(GlcNAc)_3_ (G-G-G-P)	0.081	0.877
*p*-nitrophenyl-(GlcNAc)_4_ (G-G-G-G-P)	0.004	0.089

ND, not detected; G, GlcNAc; P, *p*-nitrophenyl (pNp).

We used HPLC to analyze the hydrolysis products of (GlcNAc)_5_ by SmeChiA and SmeChiB and compared them with those of other reported fish chitinases (Table 2). Both chitinases hydrolyzed (GlcNAc)_5_ to produce (GlcNAc)_2_ + (GlcNAc)_3_, and SmeChiA hydrolyzed the second and third glycosidic bonds at the rates of 80.8% and 19.2%, respectively. In contrast, SmeChiB degraded the second and third glycosidic bonds at the rates of 29.6% and 70.4%, respectively.

When pNp-(GlcNAc)_n_ (*n* = 2–4) and (GlcNAc)_5_ were used as a substrate, both chitinases showed different substrate specificities. From these results, it became clear that SmeChiA as well as PaChiA [14], HoChiB, HoChiC [13], PtChiA [16], SmChiA, and SmChiB [17] preferentially hydrolyze the second glycosidic bond from the non-reducing end of (GlcNAc)_n_ and that SmeChiB as well as PaChiB [15] and HoChiA [13], PtChiB [16], and SmChiC [17] preferentially degrade the third glycosidic bond (Table 1 and Table 2).

**Table 2:** Reaction patterns and cleavage patterns of (GlcNAc)_5_ by SmeChiA and SmeChiB, and other fish stomach chitinases.

Fish	AFCase-1		AFCase-2		References
	Chitinase	Cleavage Patterns	Chitinase	Cleavage Patterns	
*Sardinops melanostictus*	SmeChiA (45 kDa)	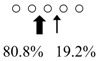	SmeChiB (49 kDa)	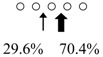	This study
*Sebastiscus marmoratus*	SmChiA (46 kDa)	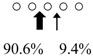	SmChiC (56 kDa)	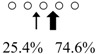	[17]
	SmChiB (52 kDa)	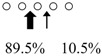			
*Parapristipoma trilineatum*	PtChiA (50 kDa)	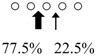	PtChiB (60 kDa)	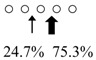	[16]
*Pennahia argentata*	PaChiA (42 kDa)	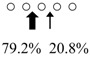	PaChiB (56 kDa)	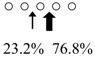	[14,15]

○ represents GlcNAc. The left side of the cleavage patterns is the non-reducing end. Thick arrows: main cleavage sites.

### 2.4. Substrate Specificities of SmeChiA toward Insoluble Substrates

We measured the substrate specificity of SmeChiA toward insoluble substrates by using crystalline α-chitin (crab shell chitin and shrimp shell chitin), crystalline β-chitin (squid pen chitin), chitin nanofibers of crystalline chitin, and non-crystalline colloidal chitin (Table 3). SmeChiA exhibited markedly high activity (33.4 U/mg) toward chitin nanofiber, which was 3- to 4.3-times higher than the activities of PtChiA [16], SmChiA, and SmChiB [17]. The second highest activity of SmeChiA was toward squid pen β-chitin (1.45 U/mg).

**Table 3:** Substrate specificities of SmeChiA toward insoluble substrates.

Substrate	Specific Activity (U/mg)
Crab shell α-chitin	0.922
Shrimp shell α-chitin	0.303
Squid pen β-chitin	1.45
Chitin nanofiber	33.4
Colloidal chitin	0.922

The activities of SmeChiA toward α-chitin, which has the most rigid crystalline structure, were as follows in descending order: crab shell chitin (0.922 U/mg) > shrimp shell chitin (0.303 U/mg). When crab shell chitin was used as a substrate, the SmeChiA activities were second highest among the other fish stomach chitinases after PaChiB [15]. SmeChiA also efficiently degraded non-crystalline colloidal chitin (Table 3). These results indicate that SmeChiA exhibits wide substrate specificity toward crystalline chitin.

### 2.5. Molecular Cloning of the Two Chitinase cDNAs

**Figure 3:**
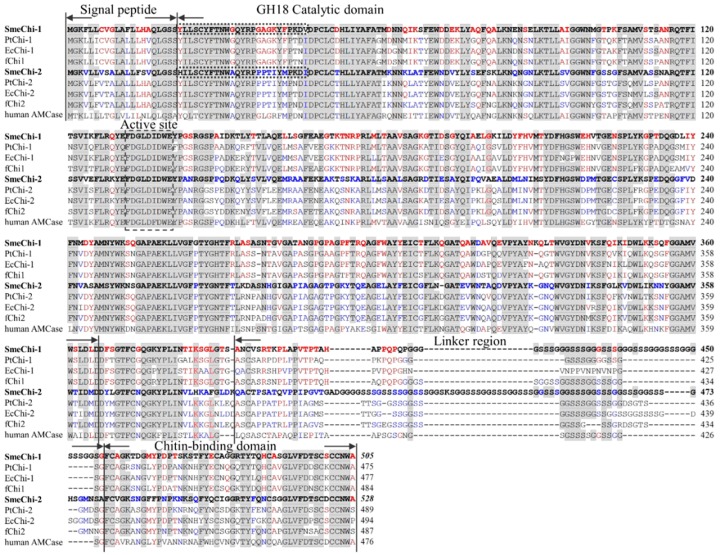
Alignment of amino acid sequences of *S. melanostictus* chitinase (SmeChi-1 and SmeChi-2), with *P. trilineatum* chitinase (PtChi-1 and PtChi-2), *Epinephelus coioides* chitinase (EcChi-1 and EcChi-2), *Paralichthys olivaceus* chitinase (fChi1 and fChi2) and human acidic mammalian chitinase (human AMCase). GenBank accession nos.: *SmeChi-1*, AB985610; *SmeChi-2*, AB985611; *PtChi-1*, AB642677; *PtChi-2*, AB642678; *EcChi-1*, FJ169895; *EcChi-2*, FJ169894; *fChi1*, AB121732; *fChi2*, AB121734; *human AMCase*, AF290004. The text surrounded by a dotted line indicates the N-terminal amino acid sequences of purified SmeChiA and SmeChiB, respectively. Identical residues of *S. marmoratus* stomach chitinase are shown with a gray background. Identical residues of SmeChi-1 only are shown with a red-letter. Identical residues of SmeChi-2 only are shown with a blue-letter.

We cloned the two cDNAs *SmeChi-1* encoding SmeChiA purified from the stomach of *S. melanostictus*, and *SmeChi-2* encoding SmeChiB. The sequences determined for the two cDNAs encoding the chitinases *SmeChi-1* and *SmeChi-2* were registered with the DNA Data Bank of Japan (DDBJ) database (accession nos. AB985610 for *SmeChi-1* and AB985611 for *SmeChi-2*). The *SmeChi-1* cDNA was cloned up to 1,543 bp and contains an ORF of 1,515 bp encoding 505 amino acids. The *SmeChi-2* cDNA was cloned up to 1,622 bp and contains an ORF of 1,584 bp encoding 528 amino acids (Figure 3).

A FASTA analysis revealed that the deduced amino acid sequences of both *SmeChi-1* and *SmeChi-2* (SmeChi-1 and SmeChi-2) showed high identities (73.9%–80.4%) to those of other fish stomach chitinases of Osteichthyes. SmeChi-1 and SmeChi-2 also showed 51.7%–65.1% identities to those of the acidic mammalian chitinase of GH family 18 chitinases of vertebrates including humans.

SmeChi-1 and SmeChi-2 contained N-terminal signal peptides, GH18 catalytic domains, linker regions, and C-terminal chitin-binding domains (Figure 3), and the linker regions of SmeChi-1 and SmeChi-2 are the longest among the fish stomach chitinases identified thus far. Parts of the amino acid sequences of the GH18 catalytic domains of both enzymes were detected as the sequence of the “DXDXE” active-site motif, which conserves sequences of GH family 18 chitinases including other fish stomach chitinases (Figure 3). In addition, the 25-residue N-terminal amino acid sequence of the GH18 catalytic domain of SmeChi-1 was completely concordant with those of purified SmeChiA and that of SmeChi-2 was also completely concordant with that of purified SmeChiB (Figure 3).

### 2.6. Tissue Expressions of *SmeChi-1* and *SmeChi-2*

**Figure 4:**
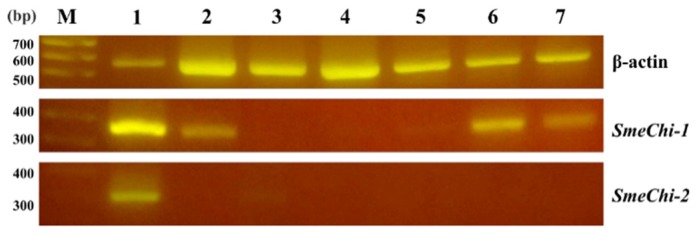
Expressions of *SmeChi-1*, *SmeChi-2*, and β-actin mRNA in tissues by RT-PCR. Lane M: markers. Lane 1: stomach. Lane 2: intestine. Lane 3: liver. Lane 4: spleen. Lane 5: kidney. Lane 6: testis. Lane 7: ovary. β-actin was used as a control.

**Figure 5:**
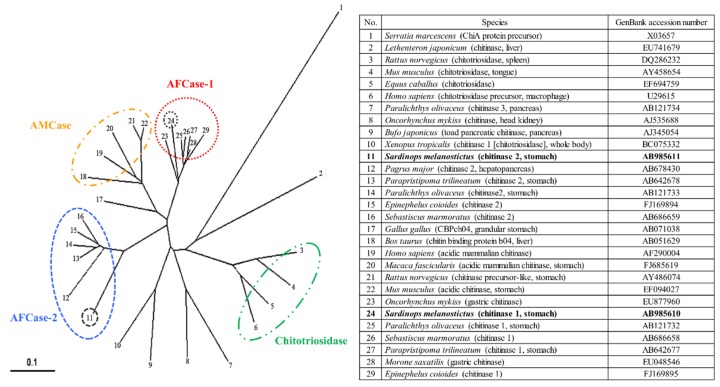
Phylogenetic tree analysis of chitinase amino acid sequences by the neighbor-joining method using ClustalW2. A bacterial chitinase, *Serratia marcescens* chitinase, was used as an outgroup. The scale bar indicates the substitution rate per residue. The black circles show SmeChi-1 and SmeChi-2 obtained in the present study.

We analyzed the expressions of *SmeChi-1* and *SmeChi-2* in different *S. melanostictus* tissues by RT-PCR using the housekeeping gene, β-actin, as a control. *SmeChi-1* mRNAs were found in the stomach, intestine, testis, and ovary (Figure 4). The *fChi1* mRNA of Japanese flounder *Paralichthys olivaceus* was found predominantly in the stomach, and at a lower level, in the testis and ovary [21], and the distribution of *SmeChi-1* mRNA was similar to that of *fChi1.* *SmeChi-2* mRNA was found in the stomach, which is concordant with the distributions of *PtChi-1* and *PtChi-2* mRNA in *P. trilineatum* [16]. As shown in Figure 5, *SmeChi-1* was expressed more strongly than *SmeChi-2* as well as *PtChi-1* [16].

### 2.7. Phylogenetic Analysis of SmeChi-1 and SmeChi-2

The phylogenetic tree analysis using SmeChi-1 and SmeChi-2 obtained in this study and those of other vertebrate chitinases revealed that SmeChi-1 and SmeChi-2 can be classified into distinct chitinase groups: SmeChi-1 was classified into fish-specific acidic fish chitinase-1 (AFCase-1), and SmeChi-2 was classified acidic fish chitinase-2 (AFCase-2) (Figure 5). *S. melanostictus* has two different chitinases (AFCase-1 and AFCase-2) with different degradation patterns expressed in the stomach, and SmeChiA and SmeChiB both have a chitin-degrading enzymatic system that efficiently degrades chitin ingested as food, as well as a previous reported fish stomach chitinases [13,14,15,16,17,22].

### 2.8. Prediction of the 3D Structural Models of SmeChi-1 and SmeChi-2

**Figure 6:**
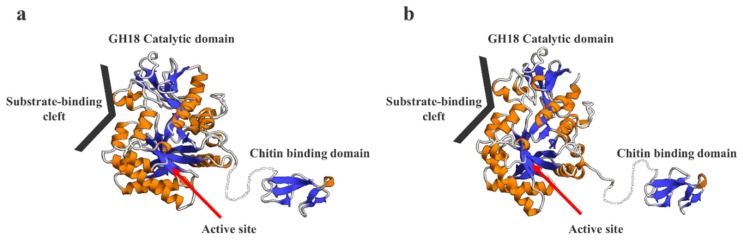
3D structure prediction models of SmeChi-1 (**a**) and SmeChi-2 (**b**). The 3D structure models were predicted using the SWISS-MODEL program (http://swissmodel.expasy.org/). The structure of the catalytic domain of acidic mammalian chitinase from *Homo sapiens* (Protein Data Bank [PDB] ID: 3FY1) and those of chitin-binding proteins from *Tachypleus tridentatus* (PDB ID: 1DQC) were used as a template.

We predicted the 3D structures of SmeChi-1 and SmeChi-2 by using the structure of the catalytic domain of acidic mammalian chitinase from *Homo sapiens* (Protein Data Bank [PDB] ID: 3FY1) and those of chitin-binding proteins from *Tachypleus tridentatus* (PDB ID: 1DQC) as a template. SmeChi-1 and SmeChi-2 had catalytic domains that consisted of a TIM-barrel (β/α)_8_-fold [8,9] structure. It is thought that this structure is a characteristic of GH18 family chitinases and that it hydrolyzes the β-1, 4 linkage of chitin by its own catalytic mechanism (Figure 6). In addition, the 3D structures of SmeChi-1 and SmeChi-2 revealed a deep substrate-binding cleft similar to that of *Bacillus circulans* chitinase A1 [32], and this cleft is needed to important in processive hydrolysis of the chitin chain [33]. The present results showed that SmeChiA efficiently degraded crystalline chitin and exhibited especially high activity toward chitin nanofibers (Table 3).

SmeChi-1 and SmeChi-2 have the longest linker regions among the fish stomach chitinases identified to date (Figure 3). SmeChiA was adsorbed by affinity columns when chitin was used as a carrier. In light of these results, it seems that SmeChiA has chitin binding ability hydrolyze chitin with moving on the surface of chitin. Our present findings also suggest that the catalytic domain of SmeChiA can hydrolyze a wide area of chitin by using the longest linker region and the chitin binding domain. This mechanism may underlie the high chitinase activity toward crystalline chitins.

## 3. Experimental Section

### 3.1. Chemicals

*p*-nitrophenyl *N*-acetylchitooligosaccharides (pNp-(GlcNAc)_n_, *n* = 1–4) and penta-*N*-Acetyl-chitopentarose (GlcNAc)_5_ was purchased from Seikagaku (Tokyo, Japan). Crab shell chitin (α-chitin) was from Tokyo Chemical Industry (Tokyo, Japan). Shrimp shell chitin (α-chitin, Chitin EX) was from Funakoshi (Tokyo, Japan). Squid pen chitin (β-chitin) was kindly provided by Kyowa Technos (Chiba, Japan). Chitin nanofiber was a generous gift from Dr. Shinsuke Ifuku (Tottori University, Tottori, Japan). Colloidal chitin was prepared according to the method of Shimahara and Takiguchi [34].

### 3.2. Purification of SmeChiA and SmeChiB

Chitinase isozymes (SmeChiA and SmeChiB) were purified from the stomach (stomach weight: 3.58 g) of *S. melanostictus* by ammonium sulfate fractionation (0%–70% saturation) and column chromatography using Chitin EX (pH 7.2), TOYOPEARL CM-650S (pH 5.5) for SmeChiA, and TOYOPEARL DEAE-650S (pH 7.2) and TOYOPEARL CM-650S (pH 4.5) for SmeChiB, according to the method of Ikeda *et al.* [14,15,16,17].

### 3.3. Chitinase Activity Assay

We assayed the chitinase activity using various substrates. First, pNp-(GlcNAc)_2_ and pNp-(GlcNAc)_3_ were used as a substrate to measure enzyme activity during the purification of chitinases. When pNp-(GlcNAc)_n_ (*n* = 1–4) was used as the substrate, the enzyme activity was assayed by the method of Ohtakara [35]. Briefly, 25 μL of enzyme solution and 10 μL of 4 mM pNp-(GlcNAc)_n_ were added to 25 μL of 0.2 M phosphate–0.1 M citrate buffer (pH 4.5) and incubated for 10 min at 37 °C. After incubation, 100 μL of 0.2 M sodium carbonate solution was added, and the absorbance of the released *p*-nitrophenol was measured at 420 nm. One unit of enzyme activity was defined as the amount of enzyme releasing 1 μmol of *p*-nitrophenol per min at 37 °C.

The hydrolysis products of (GlcNAc)_5_ produced by SmeChiA and SmeChiB and their anomer formation ratios were analyzed according to the method of Koga *et al.* [36]. Briefly, 5 μL enzyme solution and 25 μL 0.22 mM (GlcNAc)_n_ were added to 25 μL 0.1 M sodium acetate buffer (pH 4.0), and the mixture was incubated for 10 min at 25 °C. The reaction was stopped by cooling to 0 °C in an ice bath. The reaction solution was analyzed at 25 °C by high-performance liquid chromatography (HPLC) using a TSK-GEL Amide-80 column (4.6 mm dia. × 250 mm, Tosoh, Tokyo, Japan). (GlcNAc)_5_ was eluted with 70% acetonitrile solution at a flow rate of 0.8 mL/min, and the absorbance was measured at 210 nm.

When 0.5% colloidal chitin, 0.5% α- or β-chitin, or 1% chitin nanofiber was used, the enzyme activity was assayed by the method of Ohtakara [35]. Briefly, 250 μL of enzyme solution and 250 μL of substrate solution were added to 500 μL of 0.2 M phosphate–0.1 M citrate buffer (pH 4.5), and the mixture was incubated for 2 h at 37 °C with shaking. After the incubation, the reaction was stopped by boiling for 3 min. The reaction solution was centrifuged, and 375 μL of the supernatant was sampled. To measure the amount of reducing sugar produced by the enzymatic reaction, 500 μL of Schales’ reagent was added to the collected solution, and the absorbance was measured at 420 nm. The solution was then boiled for 15 min and cooled in running water. The absorbance was then measured again at 420 nm. The standard curve was prepared using authentic GlcNAc, and the absorbance was then converted into the amount of GlcNAc. One unit of enzyme activity was defined as the amount of enzyme required to degrade substrates at 37 °C and produce reducing sugars corresponding to 1 μmol GlcNAc per min.

### 3.4. Effect of pH on Chitinase Activity

When pNp-(GlcNAc)_2_ and pNp-(GlcNAc)_3_ were used as a substrate, the optimum pH was determined by assaying the enzyme activity. Briefly, the solution was incubated for 10 min at 37 °C in 0.1 M sodium acetate–0.1 M HCl buffer (pH 1.0–2.0) or 0.2 M phosphate–0.1 M citrate buffer (pH 2.5–8.0) for SmeChiA and 0.2 M phosphate–0.1 M citrate buffer (pH 2.5–8.0) for SmeChiB.

### 3.5. Protein Measurement

Protein concentrations were measured by the method of Bradford using bovine serum album as the standard [37].

### 3.6. Gel Electrophoresis

SDS-PAGE was carried out by the method of Laemmli [38] with 12.5% polyacrylamide gel (e-PAGEL, Atto, Tokyo, Japan). A sample was mixed with Ez Apply (Atto), and the mixture was heated for 5 min at 100 °C. The proteins in the gels were stained with Coomassie Brilliant Blue R-250.

### 3.7. N-Terminal Amino Acid Sequence Analysis

The N-terminal amino acid sequences were analyzed using a protein sequencer (PE Applied Biosystems 447/120A, Foster City, CA, USA).

### 3.8. Cloning of *S. melanostictus* cDNA

The sequences of all primers are presented in Supplementary Table S1. Total RNA was extracted from the stomach of *S. melanostictus* using ISOGEN II reagent (Nippon Gene, Tokyo, Japan) according to the manufacturer’s instructions. First-strand cDNA was synthesized using 500 ng total RNA and oligo dT primers with PrimeScript II Reverse Transcriptase (RNase H-free) (Takara Bio, Shiga, Japan) according to the manufacturer’s instructions. The reverse transcriptase–polymerase chain reaction (RT-PCR) was performed with primers Chi-a, Chi-b and Chi-c for *SmeChi-1* and Chi-d, Chi-e, and Chi-f for *SmeChi-2*, respectively. The first PCR was carried out with *S. melanostictus* cDNA as a template, with Chi-a and Chi-c for *SmeChi-1* and Chi-d and Chi-f for *SmeChi-2* as primers. Nested PCR was performed with the products of the first PCR as the templates, and with Chi-b and Chi-c for *SmeChi-1* and Chi-e and Chi-f for *SmeChi-2* as primers (Table S1, Figure S1).

To obtain the full-length cDNA of *SmeChi-1* and *SmeChi-2*, we performed both 3′- and 5′-RACE (rapid amplification of cDNA ends) using a gene-specific primer based on the sequence of the cDNA fragment obtained from the RT-PCR. cDNA fragments encoding the 3′ region of *SmeChi-1* and *SmeChi-2* were amplified with *S. melanostictus* cDNA as the template and the primer pairs *SmeChi-1*-1 and 3R, and *SmeChi-2*-1 and 3R, respectively.

The 5′-RACE was performed using the 5′-Full RACE Core Set (Takara Bio, Shiga, Japan) according to the manufacturer′s instructions. Initially, first-strand cDNA was newly synthesized using mRNA. For *SmeChi-1*-2 and *SmChi-2*-2 were used as the respective 5′-phosphated primers for reverse transcription in the reaction (Figure S1). cDNA fragments encoding the 5′ regions of *SmeChi-1* and *SmeChi-2* were amplified two and three times by PCR. The first PCR was performed using the cyclized first-strand cDNA as a template and the primer pairs *SmeChi-1*-3 and *SmeChi-1*-4 for *SmeChi-1*, and *SmeChi-2*-3 and *SmeChi-2*-4 for *SmChi-2*. The second PCR was performed using the first PCR products as templates and the primer pairs *SmeChi-1*-5 and *SmeChi-1*-6 for *SmeChi-1* and *SmeChi-2*-5 and *SmeChi-2*-6 for *SmeChi-2*.

Lastly, the nucleotide sequences of cDNA fragments containing full-length open reading frames (ORFs) were confirmed by PCR using specific primers (*SmeChi-1*-7 and *SmeChi-1*-8 for *SmeChi-1*, and *SmeChi-2*-7 and *SmeChi-2*-8 for *SmChi-2*) and Platinum^®^ *Pfx* DNA Polymerase (Life Technologies, Carlsbad, CA, USA).

### 3.9. Nucleotide Sequence Analysis

We subcloned the RT-PCR, 3′ RACE, and 5′ RACE amplification products into pGEM-T^®^ Easy vector (Promega, Madison, WI) according to the respective manufacturer’s instructions. We carried out A-tailing with the full-length amplification products of *SmeChi-1*, and these products were subcloned into pGEM-T Easy vector. The full-length amplification products of *SmeChi-2* were subcloned into pCR-Blunt II-TOPO^®^ vector (Life Technologies, Carlsbad, CA, USA). Sequences were determined on an ABI PRISM 3130 genetic analyzer (Applied Biosystems, Foster City, CA, USA) using the Big Dye Terminator v3.1 cycle sequencing kit (Applied Biosystems, Foster City, CA, USA).

### 3.10. Phylogenetic Analysis of SmeChi-1 and SmeChi-2

To classify the chitinases from the stomach of *S. melanostictus* among vertebrate chitinases, we constructed a phylogenetic tree based on the sequences of enzyme precursors by the neighbor-joining method using the ClustalW2 program (http://www.ebi.ac.uk/Tools/msa/clustalw2/). A bacterial chitinase (GenBank: X03657) was used as an outgroup.

### 3.11. Prediction of the 3D Structure Models of SmeChi-1 and SmeChi-2

The 3D structure models of SmeChi-1 and SmeChi-2 were predicted using the SWISS-MODEL program (http://swissmodel.expasy.org/), based on template the acidic mammalian chitinase catalytic domain in complex with methylallosamidin (PDB ID: 3fy1) for the structure of the catalytic domain and the solution structure of tachycitin, an antimicrobial protein with chitin-binding function (PDB ID: 1dqc) for the chitin-binding domain.

### 3.12. Tissue-Specific Gene Expressions of *SmeChi-1* and *SmeChi-2*

Total RNA was prepared from the stomach, intestine, hepatopancreas, pyloric appendage, kidneys, testis, and ovary of *S. melanostictus* as described in the RNA isolation section above. First-strand cDNA was prepared from the RNA isolated from each tissue as described in the RT-PCR section above. *SmeChi-1* and *SmeChi-2* were amplified using the first-strand cDNA as the template and the primer pairs *SmeChi-1*-a and *SmeChi-1*-b for *SmeChi-1*, and *SmeChi-2*-a and *SmeChi-2*-b for *SmeChi-2* (Table S1). To determine the amount of total RNA in each tissue, β-actin mRNA fragments were amplified using specific primer pairs (Table S1).

## 4. Conclusions

The linker regions of SmeChi-1 and SmeChi-2 are the longest among the fish stomach chitinases identified thus far. In addition, the 3D structures of SmeChi-1 and SmeChi-2 revealed a deep substrate-binding cleft that is needed to degrade insoluble substrates. The present results showed that SmeChiA efficiently degraded crystalline chitin and SmeChiA was adsorbed by affinity columns when chitin was used as a carrier. Our present findings also suggest that the catalytic domain of SmeChiA can hydrolyze a wide area of chitin by using the longest linker region and the chitin binding domain. This mechanism may underlie the high chitinase activity toward crystalline chitins.

## Supplementary Files

Supplementary File 1

## Abbreviations

(GlcNAc)_n_: *N*-acetylchitooligosaccharides
GlcNAc: *N*-acetyl-d-glucosamine
pNp-(GlcNAc)_n_: *p*-nitrophenyl *N*-acetylchitooligosaccharides
HPLC: high-performance liquid chromatography
RACE: rapid amplification of cDNA ends
SDS-PAGE: sodium dodecyl sulfate polyacrylamide electrophoresis
