# Supplementary material for: Stomach Chitinase from Japanese Sardine Sardinops melanostictus: Purification, Characterization, and Molecular Cloning of Chitinase Isozymes with a Long Linker
Source: Mar Drugs. 2016 Jan 20;14(1):22. doi: 10.3390/md14010022 (PMC4728518; doi:10.3390/md14010022)
Supplement: Supplementary File 1 [file marinedrugs-14-00022-s001.pdf]

Supplementary Materials: Stomach Chitinase from Japanese Sardine *Sardinops melanostictus*: Purification, Characterization, and Molecular Cloning of Chitinase Isozymes with a Long Linker

Satoshi Kawashima, Hiroki Ikehata, Chihiro Tada, Tomohiro Ogino, Hiromi Kakizaki, Mana Ikeda, Hideto Fukushima and Masahiro Matsumiya

Table S1. Primers used for PCR, RACE, and tissue expression. [1] Degenerate primers; [2] 5′-phosohorylated primers.

Primer	Sequence (5′→3′)	Purpose
Chi-a [1]	TGYTAYTTYACNAAYTGG	Primary PCR
Chi-b [1]	GAYATHGAYTGGGARTAYCC	Primary PCR
Chi-c [1]	TTCCARTARTTCATNGCRTARTC	Primary PCR
Chi-d [1]	CCNCCNACNATHTAYATGCC	Primary PCR
Chi-e [1]	GTNTTYYTNGARGARATGMG	Primary PCR
Chi-f [1]	TCYTGNGTRTAYTTNCCNGG	Primary PCR
SmeChi-1 -1	CAGGGAGACCTCATCTACTTCAAC	3′RACE
SmeChi-2 -1	AATATCCTGCCAACAGAGGGAGC	3′RACE
3R	CTGTGAATGCGACTACGAT	3′RACE
SmeChi-1 -2 [2]	GTTGAAGTAGATGAGGTCTCCCTG	5′RACE
SmeChi-1 -3	GACCTACGACTTCCATGGCTCCT	5′RACE
SmeChi-1 -4	TTGCCGAGCTCAGCGATCTG	5′RACE
SmeChi-1 -5	AGCACGTCACTGGTGAGAACA	5′RACE
SmeChi-1 -6	TCCAGAGTCGATGGTGCCCTTTC	5′RACE
SmeChi-2 -2 [2]	AGTCGATGTCCAGACCGTCAAACT	5′RACE
SmeChi-2 -3	GGAACTTTGGCTCTTCAGGATTC	5′RACE
SmeChi-2 -4	GGTCTTCAGATTTCCGTTCTGG	5′RACE
SmeChi-2 -5	AACGCTCGCCAGACCTTCATCA	5′RACE
SmeChi-2 -6	GAGTTTGCTGAACTCGCTGTAG	5′RACE
SmeChi-1 -7	ACCATGGGCAAGTTTCTCCTTTGC	Full-length ORF
SmeChi-1 -8	GAATGATCTAAAAGGATTTGCTCTATGC	Full-length ORF
SmeChi-2 -7	TACGAGGCAACCATGGGTAAAGTACT	Full-length ORF
SmeChi-2 -8	GGGAATAATTAATCTAGCACAGCCTAA	Full-length ORF
β-actin-a	GATCATGTTCGAGACCTTCAACAC	Tissue expression
β-actin-b	TCCAATCCAGACAGAGTATTTAGC	Tissue expression
SmeChi-1 -a	AGCTGCTGTCTGGCTTCGAGGCTGA	Tissue expression
SmeChi-1 -b	TTAGCGGTGGCACCAACTCCGGTGTT	Tissue expression
SmeChi-2 -a	AAATGAGGGCTGCCTTTGAGAAGGAGG	Tissue expression
SmeChi-2 -b	TTGCGTGTACTTCCCAGGAGTTCCA	Tissue expression

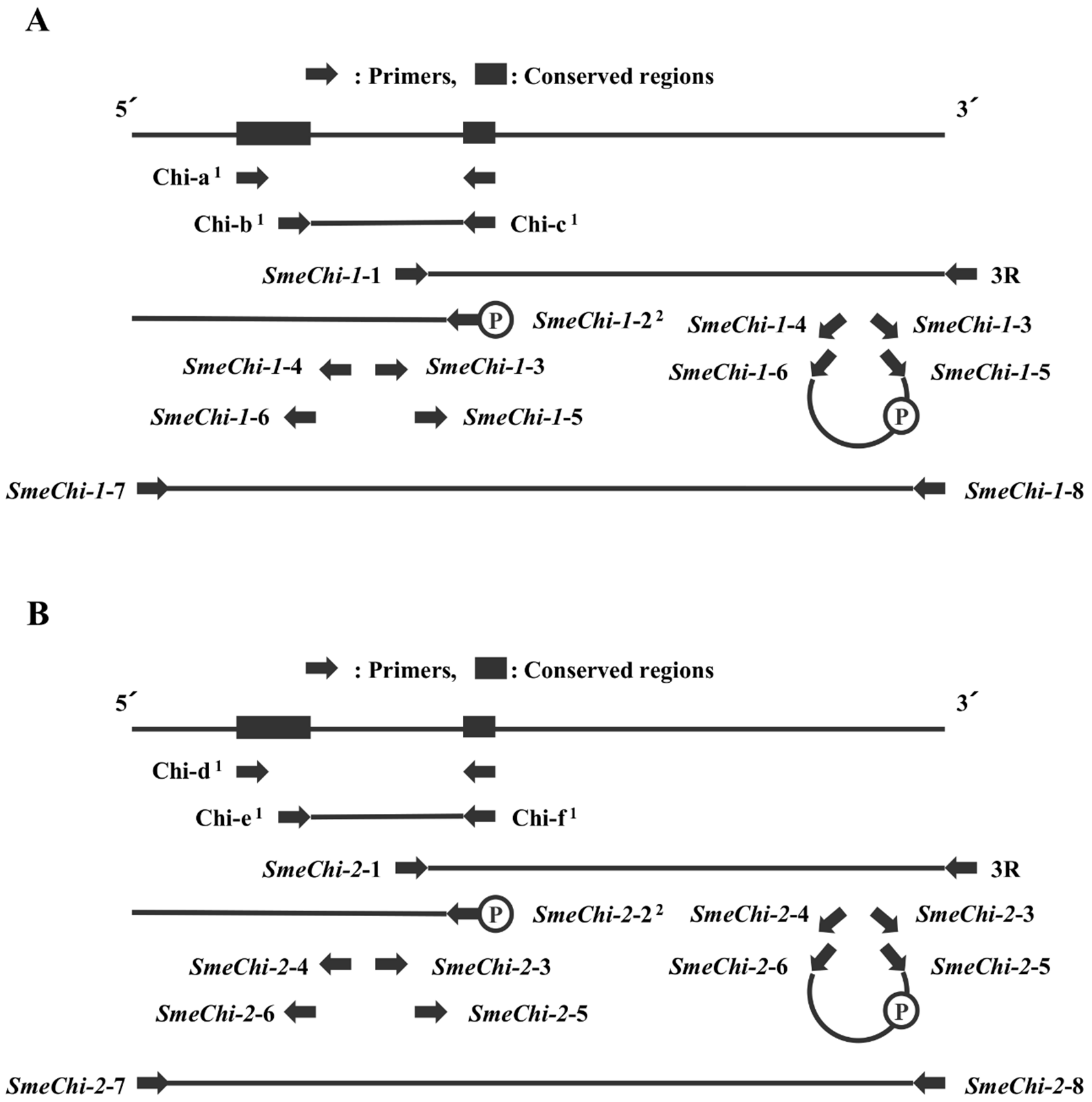

Figure S1. The position of the primers used in this study. A, *SmeChi-1*. B, *SmeChi-2*.
